# Modeling the economic and health impact of substandard uterotonics in Senegal

**DOI:** 10.1186/s12884-025-07189-9

**Published:** 2025-04-26

**Authors:** Yi-Fang Ashley Lee, Colleen R. Higgins, Petra Procter, Sara Rushwan, A. Metin Gülmezoglu, Lester Chinery, Sachiko Ozawa

**Affiliations:** 1 Division of Practice Advancement and Clinical Education, UNC Eshelman School of Pharmacy, University of North Carolina, Chapel Hill, USA; 2 Concept Foundation, Geneva, Switzerland; 3 Department of Maternal and Child Health, UNC Gillings School of Global Public Health, University of North Carolina, Chapel Hill, USA

## Abstract

**Background:**

Maternal mortality due to postpartum hemorrhage (PPH) remains a global concern especially in low- and lower-middle income countries. PPH is preventable with quality-assured uterotonics. However, substandard uterotonics pose a significant risk to PPH, and there is limited evidence available to provide quantitative estimates of their economic impact. This study aims to evaluate the impact of ensuring uterotonic quality in Senegal, highlighting the potential to lower healthcare costs, reduce maternal deaths, and contribute to achieving Sustainable Development Goals related to maternal health and Universal Healthcare Coverage.

**Methods:**

We utilized a decision tree model to estimate the economic and health impact of improving the quality of uterotonics in prevention of PPH in Senegal. We simulated women giving birth in various healthcare settings, receiving uterotonics of varied quality, and subsequent PPH-related outcomes. Data from the Senegal Demographic and Health Survey, Cochrane review, and E-MOTIVE trial informed the model. We compared scenarios with and without substandard uterotonics, along with scenarios altering uterotonic usage and care-seeking behavior.

**Results:**

Our findings indicate that utilizing quality-assured uterotonics in Senegal could lead to a notable 7–9% reduction in the overall economic burden of PPH, saving over 1 million USD annually in direct costs and long-term productivity losses. Improving the quality of uterotonics in Senegal would result in a 6–8% reduction in PPH cases, translating to over 5,000 fewer PPH cases annually. Using quality uterotonics instead of substandard ones also decreased deaths from PPH by 6–8% annually.

**Conclusions:**

This study underscores the importance of ensuring uterotonic quality to showcase significant cost savings and improvements in maternal health outcomes in Senegal. The accrued cost savings from improved maternal health outcomes related to PPH prevention and treatment would greatly benefit mothers, their families, healthcare providers, and the healthcare system. This case study offers valuable insights into improving the quality of maternal care and achieving more efficient resource allocation to advance Senegal’s progress towards Universal Health Coverage.

**Supplementary Information:**

The online version contains supplementary material available at 10.1186/s12884-025-07189-9.

## Background

Maternal mortality remains a critical global health challenge, with 95% of maternal deaths concentrated in low- and lower-middle income countries [1]. Among these countries, postpartum hemorrhage (PPH) stands out as the predominant cause, accounting for 30–50% of maternal mortalities in sub-Saharan Africa [2]. Despite being preventable and treatable, PPH affects approximately 14 million women, leading to an alarming 70,000 maternal deaths annually worldwide [3]. The World Health Organization (WHO) recommends the prophylactic administration of uterotonics during the third stage of labor, an intervention proven effective in preventing PPH [4]. However, the efficacy of these medications hinges on the quality of the uterotonics that are deployed.

Ensuring the quality of medicines is paramount to their effectiveness. Substandard medicines, defined as “authorized medical products that fail to meet either their quality standards or specifications, or both,” pose a significant threat to maternal health [5]. According to the WHO, about 1 in 10 medicines in low- and middle-income countries (LMIC) are substandard or falsified, leading to tens of thousands of deaths annually due to ineffective treatments for common diseases such as malaria and pneumonia [5]. Previous studies that sampled widely-used uterotonics such as oxytocin and misoprostol revealed that nearly half of them failed quality testing [6–9]. In Senegal, a Francophone West African country, 4–46% of uterotonic samples have been found to fail quality tests [10].

Substandard uterotonics that have low amounts of the active pharmaceutical ingredient can be ineffective at preventing PPH. Studies have identified storage conditions for oxytocin that exceed recommended temperature limits, compromising its potency [11–13]. The recent E-MOTIVE trial in Nigeria found that mothers at facilities where substandard oxytocin is administered are 20–30% more likely to hemorrhage [14]. However, the impact of substandard uterotonics on a population level remains insufficiently documented. There is a need to quantify the economic impact to strengthen the evidence base in LMICs and demonstrate the urgent need to safeguard medicine quality.

Disparities in development have resulted in unequal access to quality healthcare, notably between Anglophone and Francophone regions in Africa [15]. Francophone West Africa bears the highest disease burden on the continent, compounded by limited funding opportunities compared to its Anglophone counterparts [15–17]. New pharmaceutical products are introduced in Francophone West Africa on average 8 years after they reach the United States, while South Africa faces relatively shorter delays, ranging from 1 to 6 years [15]. These challenges underscore the obstacles confronting Francophone West Africa in securing better access to quality medicines. Despite progress in maternal health over the past two decades, Senegal fell short of meeting its Millennium Development Goal to reduce the maternal mortality ratio to 190 maternal deaths per 100,000 live births by 2015 [18, 19]. In 2020, the maternal mortality ratio was estimated at 261 maternal deaths per 100,000 live births [1, 3].

The primary objective of this study is to generate evidence on the benefits of ensuring uterotonic quality in Senegal. The effort of this study not only contributes to demonstrating the potential to avert maternal deaths but also serve as a case study, showcasing the potential financial savings from preventing illnesses that could be redirected towards Universal Health Coverage (UHC) [20, 21]. This analysis also contributes to advancing Sustainable Development Goals (SDG) [22]. Specifically, SDG 3.1 which targets the reduction in the global maternal mortality ratio to below 70 maternal deaths per 100,000 live births by 2030, and SDG 3.8, which focuses on providing access to high-quality essential healthcare services without financial risks. By showing the burden that substandard uterotonics imposes on governments, healthcare providers, and families, this study offers valuable insights for policy consideration.

## Methods

### Decision tree baseline model

We estimated the burden of substandard uterotonics in Senegal and simulated scenarios to improve uterotonic quality using a decision tree model (Supplementary Fig. 1). The decision tree model starts with an annual number of women giving birth in Senegal [23]. Using data from the Demographic and Health Survey for Senegal in 2019, births are distributed to take place at public hospitals, primary health centers, private hospitals, or at home (Table 1) [24]. Information on uterotonic utilization and costs were gathered from local key opinion leaders with expertise in obstetrics and gynecology, who provided insights on clinical practice patterns and cost structures at different facility types. Patient cost estimates for deliveries that were complicated by PPH were also collected, stratified by delivery method (vaginal or cesarean) and facility type. The model simulated the number of vaginal deliveries at primary health centers and homes, and vaginal or cesarean section deliveries at public or private hospitals. Home deliveries constituted 5% of deliveries in urban areas and 31% in rural areas [24], where it was assumed that no uterotonics were utilized based on insights from key opinion leaders.

**Table 1:** Key model inputs and sources

Parameter variable	Unit	Value (Uncertainty range)		Source
		Senegal		
**Demographics**				
Total population	People	17,316,449		UNPD 2022 ﻿^23^
Birth rate	Per 1,000 people	33		UNPD 2022 23
Maternal mortality ratio	Per 100,000 live births	261		World Bank 2020 3
Life expectancy at birth, female	Years	69		UNPD 2022 23
Mean age at maternal death	Years	29.5		Assumption Kodio et al. 29
GDP per capita	USD	$1,598.70		World Bank 2022 28
Mothers’ residence by rurality				
Urban	%	30		DHS 2019 24
Rural	%	70		
**Care-seeking locations and birth methods by rurality**				
Urban				
Public Hospital and Vaginal birth	%	10		DHS 2019 24
Public Hospital and C-section	%	4		
Public PHC and Vaginal birth	%	75		
Private Hospital and Vaginal birth	%	6		
Private Hospital and C-section	%	1		
Home	%	5		
Rural				
Public Hospital and Vaginal birth	%	3		DHS 2019 24
Public Hospital and C-section	%	2		
Public PHC and Vaginal birth	%	62		
Private Hospital and Vaginal birth	%	1		
Private Hospital and C-section	%	0		
Home	%	31		
Proportion of deliveries with PPH	%	40		Assumption based on Tort et al. 27
Proportion of referrals among severe PPH from home and PHC	%	18		Dieme et al. 26
**Utilization of Uterotonics**				
Facility				
Oxytocin	%	34		Assumption based on KOL
Oxytocin and Misoprostol	%	66		
No uterotonics given	%	0		
Home				
No uterotonics given	%	100		Assumption
**Proportion of substandard uterotonics**				
Oxytocin		Senegal	Côte d’Ivoire	
Public sector	%	5.3	54	Mati et al. 10
Private sector	%	4.2		
Misoprostol				
Public sector	%	45.5	4	Mati et al. 10
Private sector	%	25		
Home	%	38		Assumption based on Mati et al. 10
**Risk of health outcomes**				
Oxytocin		Vaginal birth	C section	
PPH ≥ 500 ml		0.12 (0.10–0.15)	0.6 (0.57–0.63)	Gallos et al. 25
PPH ≥ 1000 ml		0.03 (0.02–0.04)	0.13 (0.13–0.14)	
Oxytocin + Misoprostol				
PPH ≥ 500 ml		0.09 (0.07–0.11)	0.42 (0.35–0.52)	
PPH ≥ 1000 ml		0.03 (0.02–0.03)	0.12 (0.09–0.13)	
Misoprostol				
PPH ≥ 500 ml		0.13 (0.12–0.15)		
PPH ≥ 1000 ml		0.04 (0.03–0.04)		
Heat-stable carbetocin				
PPH ≥ 500 ml		0.09 (0.08–0.10)	0.44 (0.39–0.48)	
PPH ≥ 1000 ml		0.03 (0.02–0.03)	0.12 (0.10–0.13)	
No prophylactic uterotonics				
PPH ≥ 500 ml		0.24 (0.19–0.29)		
PPH ≥ 1000 ml		0.05 (0.04–0.06)		
Risk of PPH using substandard vs. quality-assured uterotonics				
PPH ≥ 500 ml	Risk Ratio	1.29		Gallos et al. 14
PPH ≥ 1000 ml	Risk Ratio	1.26		
Proportion of women with PPH receiving additional uterotonic treatments				
With quality-assured uterotonics	%	58		Assumption based on Gallos et al. 14
With substandard uterotonics	%	73		
Proportion of women with PPH receiving blood transfusion				
With quality-assured uterotonics	%	19		Assumption based on Gallos et al. 14
With substandard uterotonics	%	38		
Proportion of postpartum surgery among vaginal births with severe PPH	%	20		KOL Opinion
**Costs**				
**Public hospital**				
Vaginal births				Estimation by KOL
No PPH	USD	20.44		
Mild PPH	USD	30.97		
Severe PPH without surgery	USD	43.16		
Severe PPH with surgery	USD	66.76		
C-sections				
No PPH	USD	53.84		
Mild PPH	USD	69.94		
Severe PPH without surgery	USD	77.06		
Severe PPH with surgery	USD	100.66		
**PHC**				
Vaginal births				
No PPH	USD	20.44		
Mild PPH	USD	24.97		
Severe PPH without surgery	USD	31.40		
**Private hospital**				
Vaginal births				
No PPH	USD	213.73		
Mild PPH	USD	224.91		
Severe PPH without surgery	USD	289.83		
Severe PPH with surgery	USD	1,065.83		
C-sections				
No PPH	USD	788.36		
Mild PPH	USD	806.03		
Severe PPH without surgery	USD	861.83		
Severe PPH with surgery	USD	1,605.83		

C-section = Cesarean section; DHS = Demographic and Health Survey; GDP = Gross Domestic Product; KOL = Key Opinion Leader; PHC = primary health center; PPH = postpartum hemorrhage; UNPD = United Nations Population Division; USD = United States dollars; WHO = World Health Organization

All births in health facilities or hospitals are assumed to receive some prophylactic uterotonic in our model. The uterotonic regimens in the baseline model are (1) oxytocin together with misoprostol or (2) oxytocin alone. Delivering mothers could receive substandard or quality-assured uterotonics, resulting in four simulated options for uterotonic use at facilities: (1) substandard oxytocin with misoprostol, (2) substandard oxytocin alone, (3) quality oxytocin with misoprostol, or (4) quality oxytocin alone. The proportion of substandard medicines was taken from a post-market surveillance study conducted by the WHO on uterotonic quality in Senegal [10].

After uterotonic use, delivering mothers in the model face the probability of PPH occurring [25]. A PPH case is defined by bleeding ≥ 500 ml whereas severe PPH is defined as bleeding ≥ 1,000 ml. Further complications and health outcomes including blood transfusions, the use of more uterotonic treatment, surgery, and death may follow PPH, based on data compiled in a network meta-analysis of uterotonics for prevention of PPH [25]. The probability of dying from PPH was applied only to the branch where ≥ 1,000 ml of blood loss occurred, and was calculated to align with the maternal mortality rate for Senegal [3]. The probability of needing surgery after a PPH occurrence was collected from key opinion leaders in Senegal. For home births in the model, no prophylactic uterotonics are given and this leads to higher rates of PPH and poorer health outcomes [25]. Home births and births at primary health facilities were applied a probability of being referred to a higher level hospital facility after PPH [26].

The branches of the decision tree with substandard uterotonics lead to increased risks of PPH occurring, which then results in higher probabilities of further uterotonic treatment, blood transfusion, surgery, and death from PPH. The increase in PPH occurrence due to poor uterotonic quality is applied as a multiplier to the risk of PPH, abstracted from the E-MOTIVE trial data in Nigeria [14].

### Scenarios

To estimate the burden of substandard uterotonics in Senegal, we ran the baseline decision tree model using data on the level of poor quality uterotonics in the country. Then a scenario where all uterotonics were standard (Scenario 1) was implemented, removing the “Substandard Oxytocin” and “Substandard Misoprostol + Oxytocin” branches from the decision tree.

Further scenarios provide context and compare different solutions to improve medicine quality. One scenario modeled greater use of effective uterotonics by examining the effect of all births delivered at facilities (public or private hospitals and primary care centers) receiving preventative uterotonics of misoprostol with oxytocin where every dose is quality-assured (Scenario 2). This means that no one uses oxytocin alone, and there are no substandard oxytocin or substandard misoprostol. Another scenario examined the use of quality-assured heat-stable carbetocin as the only uterotonic being administered (Scenario 3).

Four additional scenarios simulated improvements in PPH outcomes through home births. The first two scenarios estimated the impact of distributing misoprostol for all births occurring at home, where it is currently rare for a uterotonic to be utilized. In Scenario 4, the quality of the misoprostol used was determined based on the weighted average failure rate, calculated by sample size, as reported in the literature (Table 1) [10]. In Scenario 5, all misoprostol given at home births were to be quality-assured. Then, two scenarios were run in which births only occurred at healthcare facilities and none happened at home. This meant that the quarter of women who birthed at home were instead simulated to go to a healthcare facility where they received prophylactic uterotonics. In Scenario 6, the quality of misoprostol and oxytocin was at the levels reported for public and private sectors in the literature (Table 1), where only the place of birth was changed. In Scenario 7, all women birthed at a healthcare facility and the quality of all uterotonics was improved.

### Model outcomes

The model simulated the number of births in Senegal with the health consequences for mothers receiving quality, substandard, or no uterotonics and tracked the costs associated with those health outcomes. The health outcomes tracked throughout the model were the number of PPH cases and severe PPH cases. Not all cases of PPH are diagnosed in health facilities, resulting in differing estimates of PPH between maternal surveillance data and clinical trials. To account for this, numbers of diagnosed and undiagnosed cases with ≥ 500 ml and ≥ 1,000 ml blood loss were tracked separately in the model and 40% of cases were estimated to be diagnosed [27]. Health complications and consequences of PPH were tracked, including the number of uterotonic treatments used to treat a PPH case, the number of blood transfusions, and the number of deaths resulting from PPH. The costs in USD were estimated for direct healthcare utilization of public and private facilities. Additionally, the long-term productivity losses due to death from PPH were calculated using years of life lost from maternal death from PPH multiplied by the annual GDP per capita, discounted at 3% [28]. The years of life lost were calculated using the average age of maternal death in Senegal [29]. These outcomes were tracked for each scenario and results were subtracted from baseline model results. To estimate the benefits of assuring uterotonic quality within the UHC framework, outcomes of the model were calculated from varying perspectives – those of the family, the healthcare providers, and the government.

### Sensitivity analysis

A probabilistic sensitivity analysis was conducted by ranging the model inputs 1,000 times based on input distributions. The rates of facility and mode of delivery, uterotonic use, and quality were ranged using a beta distribution. The costs were ranged using gamma distributions. The risk ratios of PPH and severe PPH were ranged with triangular distributions. The results represent the mean for the baseline model and each scenario, along with the inter quartile range (IQR). Additionally, an alternative analysis was conducted by incorporating the quality of medicine data from Côte d’Ivoire [10], simulating the potential impact of variations in medicine quality on the model outcomes. These simulations provided a range of estimates to reflect the uncertainties in uterotonic quality input parameters.

## Results

The annual health and economic burden of PPH in Senegal is shown in Table 2 under the Baseline model. The model estimated 86,542 cases of PPH, where 22,306 cases were diagnosed. Of the total occurrences of PPH in Senegal, 24.5% (21,152 cases) had blood loss greater than 1,000 ml, with 6,386 of those being diagnosed. The Senegal model estimated 32,250 PPH cases that required additional uterotonics and 14,408 PPH cases that needed blood transfusions annually. It was estimated that PPH resulted in 447 (IQR 411–480) deaths annually in Senegal. The economic burden of PPH was estimated at $19,499,322 (IQR $17,915,171 - $20,943,962) each year. The public sector was projected to incur $1,774,990 per year in direct costs.

**Table 2:** Annual health and economic burden of postpartum hemorrhage in Senegal comparing baseline to no substandard uterotonics

	Baseline	IQR		No substandard uterotonics	Difference	IQR of difference		Diff %
**Health outcomes**								
PPH ≥ 500 ml	86,542	84,046	88,726	81,540	-5,002	-5,817	-4,137	-6%
PPH ≥ 1,000 ml	21,152	20,429	21,871	19,769	-1,383	-1,726	-1,023	-7%
PPH ≥ 500 ml diagnosed	22,306	21,521	23,041	20,275	-2,031	-2,429	-1,606	-9%
PPH ≥ 1,000 ml diagnosed	6,386	6,137	6,657	5,823	-563	-743	-366	-9%
Additional uterotonic treatment	35,250	34,035	36,437	29,331	-5,918	-6,493	-5,240	-17%
Blood transfusions	14,408	13,846	14,938	9,643	-4,765	-5,156	-4,359	-33%
Deaths due to PPH	447	411	480	418	-28	-74	17	-6%
**Economic outcomes**								
Total economic burden of PPH	$19,499,322	$17,915,171	$20,943,962	$18,221,486	-1,277,836	-2,986,716	494,788	-7%
Total OOP costs public	$1,774,990	$956,429	$2,103,519	$1,615,355	-159,634	-188,828	-80,720	-9%
Total OOP costs private	$833,766	$702,163	$939,894	$787,498	-46,268	-95,959	3,656	-6%
Long term productivity loss	$16,890,566	$15,557,186	$18,150,050	$15,818,633	-1,071,933	-2,808,936	648,216	-6%

Diff = Difference; IQR = Interquartile range; OOP: out-of-pocket; PPH: postpartum hemorrhage; USD: United States Dollars

In comparison, providing only quality-assured uterotonics to mothers in Senegal resulted in 6% fewer PPH cases, which was 5,002 (IQR 4,137–5,817) fewer each year. This reduced the number of additional uterotonic treatments by 5,918 (IQR 5,240–6,493) and the number of blood transfusions by 33% annually. Using quality-assured uterotonics instead of substandard ones resulted in an average of 6% decrease in deaths from PPH across all model runs. This resulted in a decrease in the total economic burden of PPH, saving over 1.2 million USD in direct costs and long-term productivity losses every year.

Table 3 presents the scenario results on the annual health and economic burden of PPH in Senegal. The scenario where facility births received both quality oxytocin and misoprostol as the only prophylactic uterotonic (Scenario 2) resulted in a 13% reduction in PPH, including 2,146 fewer PPH cases annually with 1,000 ml or more blood loss. The model estimated that 9,603 fewer uterotonics were used as additional treatments for PPH, 5,938 fewer blood transfusions were needed, and an average of 45 deaths were averted using adequate quality oxytocin and misoprostol. The resulting economic costs were over $2 million lower than in baseline.

**Table 3:** Scenario results on the annual health and economic burden of postpartum hemorrhage in Senegal

	PPH ≥ 500 ml	PPH ≥ 1000 ml	Additional uterotonic treatment	Blood transfusions	Deaths due to PPH	Total economic burden of PPH	Total OOP costs public	Total OOP costs private	Long term productivity loss
**0. Baseline estimate**	86,542	21,152	35,250	14,408	447	$19,499,322	$1,774,990	$833,766	$16,890,566
**1. No substandard uterotonics**	81,540	19,769	29,331	9,643	418	$18,221,486	$1,615,355	$787,498	$15,818,633
Difference	-5,002	-1,383	-5,918	-4,765	-28	-$1,277,836	-$159,634	-$46,268	-$1,071,933
Difference %	-6%	-7%	-17%	-33%	-6%	-7%	-9%	-6%	-6%
**2. Quality misoprostol + oxytocin for facility births**	75,117	19,005	25,647	8,470	402	$17,273,438	$1,408,674	$678,574	$15,186,190
Difference	-11,425	-2,146	-9,603	-5,938	-45	-$2,225,883	-$366,315	-$155,192	-$1,704,376
Difference %	-13%	-10%	-27%	-41%	-10%	-11%	-21%	-19%	-10%
**3. Quality heat-stable carbetocin for facility births**	76,250	19,334	26,277	8,669	408	$17,619,801	$1,463,591	$711,814	$15,444,396
Difference	-10,292	-1,818	-8,972	-5,738	-38	-$1,879,521	-$311,398	-$121,952	-$1,446,170
Difference %	-12%	-9%	-25%	-40%	-9%	-10%	-18%	-15%	-9%
**4. Misoprostol at reported level of quality for home births**	74,344	20,132	35,009	14,231	426	$18,701,065	$1,766,432	$832,082	$16,102,551
Difference	-12,198	-1,020	-241	-177	-21	-$798,257	-$8,557	-$1,685	-$788,015
Difference %	-14%	-5%	-1%	-1%	-5%	-4%	0%	0%	-5%
**5. Quality misoprostol for home births**	72,005	19,526	34,888	14,139	412	$18,168,530	$1,762,548	$833,887	$15,572,094
Difference	-14,537	-1,626	-362	-269	-35	-$1,330,792	-$12,441	$121	-$1,318,471
Difference %	-17%	-8%	-1%	-2%	-8%	-7%	-1%	0%	-8%
**6. All deliveries happen in facilities**	70,632	19,117	44,603	18,114	405	$18,475,308	$2,229,428	$951,006	$15,294,874
Difference	-15,910	-2,035	9,353	3,706	-42	-$1,024,014	$454,438	$117,239	-$1,595,692
Difference %	-18%	-10%	27%	26%	-9%	-5%	26%	14%	-9%
**7. All deliveries happen in facilities with quality-assured uterotonics**	64,110	17,370	36,938	11,933	367	-$16,799,964	$2,023,821	$896,540	$13,879,602
Difference	-22,431	-3,782	1,689	-2,475	-80	-$2,699,358	$248,832	$62,774	-$3,010,964
Difference %	-26%	-18%	5%	-17%	-18%	-14%	14%	8%	-18%

OOP: out-of-pocket; PPH: postpartum hemorrhage

Scenario descriptions: (1) No substandard uterotonics: The uterotonics provided at healthcare facilities are quality assured. (2) Quality misoprostol + oxytocin for facility births: All women in facilities receive both misoprostol and oxytocin, and they are quality assured. (3) Quality heat-stable carbetocin for facility births: All women in facilities receive heat-stable carbetocin and it is quality assured. (4) Misoprostol at reported level of quality for home births: Home birthing women utilize misoprostol at reported level of quality. (5) Quality misoprostol for home births: Home birthing women utilize quality-assured misoprostol. (6) All deliveries happen in facilities: All women are modeled to utilize facility care. The use and quality of uterotonics in facilities remain as baseline. (7) All deliveries happen in facilities with quality-assured uterotonics: All women are modeled to utilize facility care. The use of uterotonics in facilities remains at baseline. The uterotonics provided are quality-assured.

Using heat-stable carbetocin at facility births (Scenario 3) had a comparable impact on PPH outcomes, reducing PPH by 10,292 (12%) cases and severe bleeding by 1,818 cases annually. Additional uterotonics were used in 25% fewer cases in this scenario and blood transfusions went down by 40% compared to baseline. The heat-stable carbetocin scenario resulted in 38 fewer deaths from PPH and reduced the economic impact by $1.9 million annually.

In comparison to baseline where home births did not receive any PPH preventative medication, providing misoprostol at current levels of quality (Scenario 4) resulted in a reduction of 14% of PPH cases (12,198 cases) each year. Going further and providing quality-assured misoprostol at home births (Scenario 5) saved an additional 3% of cases, resulting in a 17% (14,537 cases) decrease. However, misoprostol at home birth was less impactful at preventing PPH deaths than in other scenarios, where the model estimated 21 fewer deaths (5% reduction) annually with misoprostol at current quality levels and 35 fewer deaths (8% reduction) every year when misoprostol was quality-assured.

If all home births were to be delivered in a facility where mothers would receive either a combination of oxytocin with misoprostol or oxytocin alone, at current levels of quality (Scenario 6), PPH occurrences would reduce by 15,910 (18% reduction) annually and more lives would be saved. While this scenario decreased the overall economic impact by reducing long-term productivity losses due to death, direct costs at public and private health facilities increased from having more facility births. The final scenario which combines quality and access where all mothers are birthing in a facility with access to quality oxytocin and misoprostol (Scenario 7) estimated that 26% (22,431) of PPH cases would be averted annually. The number of additional uterotonic treatments increased in this scenario because more people are being treated in the facility. However, since prophylactic medicines are quality-assured, it did not increase as much as in the previous scenario. Similarly, direct costs to patients increased in Scenario 7, but only by 14% in public and 8% in private facilities compared to increases of 26% in public and 14% in private facilities in the previous scenario where medicine quality was not addressed.

Taking a UHC perspective, we examined the annual impact of poor quality uterotonics on families, healthcare providers and the government (Table 4). Annually in Senegal, we modeled that 144,381 mothers receive substandard uterotonics when giving birth. Of mothers who experienced PPH, we estimated that 19,482 were given substandard uterotonics. These PPH cases affect whole families, where mothers are hospitalized for longer, and where families pay more out-of-pocket, estimated at $205,902 annually in Senegal for PPH care resulting from substandard uterotonic use. Healthcare providers are using more of their time and hospital resources, such as available blood stock, to treat cases of PPH resulting from uterotonics that do not work as expected. Healthcare providers are estimated to provide 4,765 blood transfusions annually to mothers who have received substandard uterotonics. As policy makers look to expand UHC, ensuring uterotonic quality would mean providing better quality uterotonics to 23% of all PPH cases, which could potentially avert 6% of PPH deaths annually.

**Table 4:** Outcomes related to Universal Health Coverage: Annual burden of substandard uterotonics in Senegal

Perspective	Description	Estimate
Families	Number of mothers receiving substandard uterotonics	144,381
	Number of cases of postpartum hemorrhage receiving substandard uterotonics	19,482
	Number of cases of severe postpartum hemorrhage receiving substandard uterotonics	5,542
	Out-of-pocket costs from additional treatments, blood transfusions, and longer hospitalizations due to substandard uterotonics (US$)	205,902
	Number of additional maternal deaths from using substandard uterotonics	28
Healthcare Providers	Number of additional blood transfusions from using substandard uterotonics	4,765
Governments	% receiving substandard uterotonics among postpartum hemorrhage cases	23%
	% receiving substandard uterotonics among severe postpartum hemorrhage cases	26%
	% of additional maternal deaths from using substandard uterotonics	6%

In an uncertainty analysis ranging the quality of uterotonics using data from Côte d’Ivoire as the baseline, ensuring high-quality uterotonics for mothers in Senegal was estimated to reduce total PPH cases by approximately 8% (6,816 fewer cases), maternal deaths due to PPH by 8% (37 fewer deaths), and the total economic burden by $1,703,431 (9%). Of the total savings, $196,439 (11% reduction) came from out-of-pocket expenses in the public sector, $108,315 (12% reduction) from the private sector, and $1,398,677 (8% reduction) from productivity losses.

## Discussion

This study underscores the importance of ensuring the quality of uterotonics in Senegal, which will result in economic benefits of improved maternal health outcomes. Incorporating uncertainties in the medicine quality data [10], our analysis estimates that employing quality-assured uterotonics could lead to a 6–8% reduction in PPH cases and a 7–9% decrease in the economic burden of PPH. The cost savings accrued from better maternal health outcomes associated with preventing and treating PPH can be substantial for mothers, their families, and the country. Assuring the quality of uterotonics is essential as it would improve the quality of maternal care and facilitate more efficient allocation of resources.

Unnecessary treatment expenses can be reduced by providing patients with quality medicines from the beginning. Using quality prophylactic uterotonics reduces the necessity down the line for treatments associated with PPH, which results in healthcare providers spending less time treating patients and fewer medical resources used up. Considering medicine quality is also essential for UHC expansion. As governments work to provide financial protection for a wider range of essential medicines, ensuring the quality of those medicines is necessary to produce the most effective outcomes for patients and economic benefits to the payor and health system. If the quality of medicines is not regulated when expanding UHC, more patients may have access to medicines, but more patients will also have access to substandard and falsified medicines that may be ineffective or harmful.

Despite the global presence of substandard and falsified medical products, there is a significant gap in reporting medicines quality data originating from Francophone West Africa. From a recent systematic review of studies on uterotonic quality, only 1 out of 19 studies were found in Francophone countries, while 12 of the 19 studies from Anglophone countries were conducted recently [6]. The 2022 post-market surveillance conducted by the WHO in Senegal and Côte d’Ivoire reveals a concerning discrepancy in the quality of misoprostol and oxytocin [10]. Specifically, misoprostol samples in Senegal failed quality testing more often than oxytocin, particularly within public sectors where nearly half of the samples failed [10]. In contrast, in Côte d’Ivoire, oxytocin samples failed quality testing at a higher rate than misoprostol [10]. West African countries face similar challenges in maintaining reliable supply systems, especially in rural and suburban areas [30]. The limited data on medicine quality does not offer a comprehensive picture of the actual situation, and more surveillance data needs to be collected and shared.

The observed quality of uterotonics is alarming given the life-threatening nature of PPH in resource-constrained settings. In Senegal, where access to adequate obstetric care and blood transfusions is limited, PPH transforms from a preventable and manageable condition to one with a high risk of death. The high maternal mortality ratio (MMR) in low-income countries exemplifies the inequalities in access to quality health services. Specifically, the MMR in 2020 for Senegal was reported at 261 maternal deaths per 100,000 live births, in contrast to the mere 12 maternal deaths per 100,000 live births in high-income countries [1, 3]. Moreover, Francophone Africa faces a disproportionate burden of diseases compared to its non-Francophone counterparts [16]. This disparity is largely attributed to the fragility of health care systems and services within Francophone African countries. Senegal, for example, has few fully functioning basic emergency obstetric care facilities due to personnel scarcity [31]. Therefore, our study underscores the urgent need to strengthen the quality of uterotonics to help protect women giving birth in Senegal and prevent the need for additional care to manage PPH.

While oxytocin remains the first-line choice of uterotonics and is widely used to prevent PPH, its heat sensitivity and stringent temperature requirements during manufacturing, transportation, and storage pose significant challenges especially in LMICs [32, 33]. Reports and studies have documented the deterioration of oxytocin quality outside of the cold chain [6, 7, 9, 11]. In settings where the quality of oxytocin cannot be guaranteed, alternative uterotonics, such as heat-stable carbetocin, have been recommended by the WHO [25, 33]. A network meta-analysis indicates that regimens such as oxytocin plus misoprostol and stand-alone heat-stable carbetocin may be more effective than oxytocin alone [25]. Furthermore, studies have shown that heat-stable carbetocin can be a cost-effective option under certain circumstances [34–36]. In this study, we demonstrate the potential health and economic benefits of expanding access to quality-assured uterotonic options, including heat-stable carbetocin, over the current use of substandard oxytocin and misoprostol in Senegal. These findings support the consideration of heat-stable carbetocin as part of a broader strategy to improve uterotonic quality in resource-limited settings. However, it is important to note that heat-stable carbetocin is currently indicated only for PPH prevention and cannot be used for labor induction or augmentation. Therefore, ensuring the availability of high-quality oxytocin through improved cold-chain infrastructure remains essential for maternal care.

Misoprostol is recommended by the WHO for PPH prevention and included in the WHO’s Essential Medicines List [37]. While a network meta-analysis indicates that a stand-alone misoprostol regimen is associated with lower efficacy than oxytocin [25], misoprostol is particularly suitable in low-resource settings. Misoprostol does not require refrigeration, is available in tablet form, and can be administered at the community level. In a previous study, misoprostol was compared with oxytocin and shown to be more cost-effective in settings with inadequate maternal health care, such as rural Senegal [38]. Our model simulates two potential scenarios where misoprostol is used in all home births and demonstrates promising benefits for PPH prevention. However, we only see the full benefit of expanding access to misoprostol when the quality of medicine is guaranteed.

Our study has a number of limitations. All models are inevitably constrained by the quality and availability of data inputs. There are gaps in available data in Senegal related to the quality of uterotonics and out-of-pocket costs, as well as specific data on the utilization of additional uterotonic treatments, blood transfusions, and postpartum surgery. Specifically, the WHO survey on uterotonic quality relies on a limited number of samples, which may not fully capture the uterotonic quality landscape in Senegal. To address this, we conducted sensitivity analyses incorporating uterotonic quality data from Côte d’Ivoire, given that many of the same distributors operate across both countries. Our model assumes the independent effect of poor-quality misoprostol and oxytocin and employs combined probabilities of receiving substandard quality uterotonics. While the effects of substandard uterotonics on PPH, subsequent treatment, and mortality remain inadequately studied, we incorporated estimates from the recent E-MOTIVE trial in Nigeria [14]. Additionally, our model does not account for the costs of implementing interventions, such as strengthening cold-chain transportation for oxytocin, distributing misoprostol at the community level, or deploying heat-stable carbetocin, due to a lack of available data. The risk factor of anemia was not incorporated in our model, as there are no quantitative data documenting the effects of substandard medicine quality on anemic birthing women. Ergometrine was not incorporated in this analysis as it is not the recommended and routinely used uterotonic for PPH prevention in Senegal, and no data is currently available on the quality of ergometrine in Senegal. We recognize the scarcity of data to account for population heterogeneity in Senegal, where health care resources are disparately distributed across geographical regions. To mitigate these issues, we engaged national key opinion leaders and a steering committee to guide data inputs for uterotonic utilization and costs, providing valuable insights and context for data assumptions. The model underwent calibration and validation against existing data. Sensitivity and scenario analyses were conducted to provide a comprehensive range around the inputs and assess the impact of improving the quality of uterotonics in comparison to other potential uterotonic options under the current standard of care.

## Conclusion

Our study not only highlights the financial and medical resources that could be saved in Senegal by ensuring the quality of uterotonics, but also emphasizes that promoting better maternal outcomes provides benefits for families, the health system, and society. Further country-specific data and evidence in the context of PPH is essential to bolster tailored interventions to ensure uterotonic quality for individual nations. Addressing these challenges is imperative for advancing UHC, reducing maternal mortality, and fostering sustainable improvements in maternal health outcomes.

## Electronic supplementary material

Below is the link to the electronic supplementary material.

Supplementary Material 1

## Data Availability

All data used during this study are included in this published article.

## Abbreviations

IQR: Inter quartile range
LMICs: Low-and-middle income countries
MMR: Maternal mortality rate
PPH: Post-partum hemorrhage
SDG: Sustainable Development Goals
UHC: Universal health coverage
WHO: World Health Organization
