# Supplementary material for: Modeling the economic and health impact of substandard uterotonics in Senegal
Source: BMC Pregnancy Childbirth. 2025 Apr 26;25:510. doi: 10.1186/s12884-025-07189-9 (PMC12032821; doi:10.1186/s12884-025-07189-9)
Supplement: Supplementary file 1 — Supplementary Material 1 [file 12884_2025_7189_MOESM1_ESM.docx]

**Modeling the Economic and Health Impact of Substandard Uterotonics in Senegal**

**Supplemental Figure 1. Decision tree model**


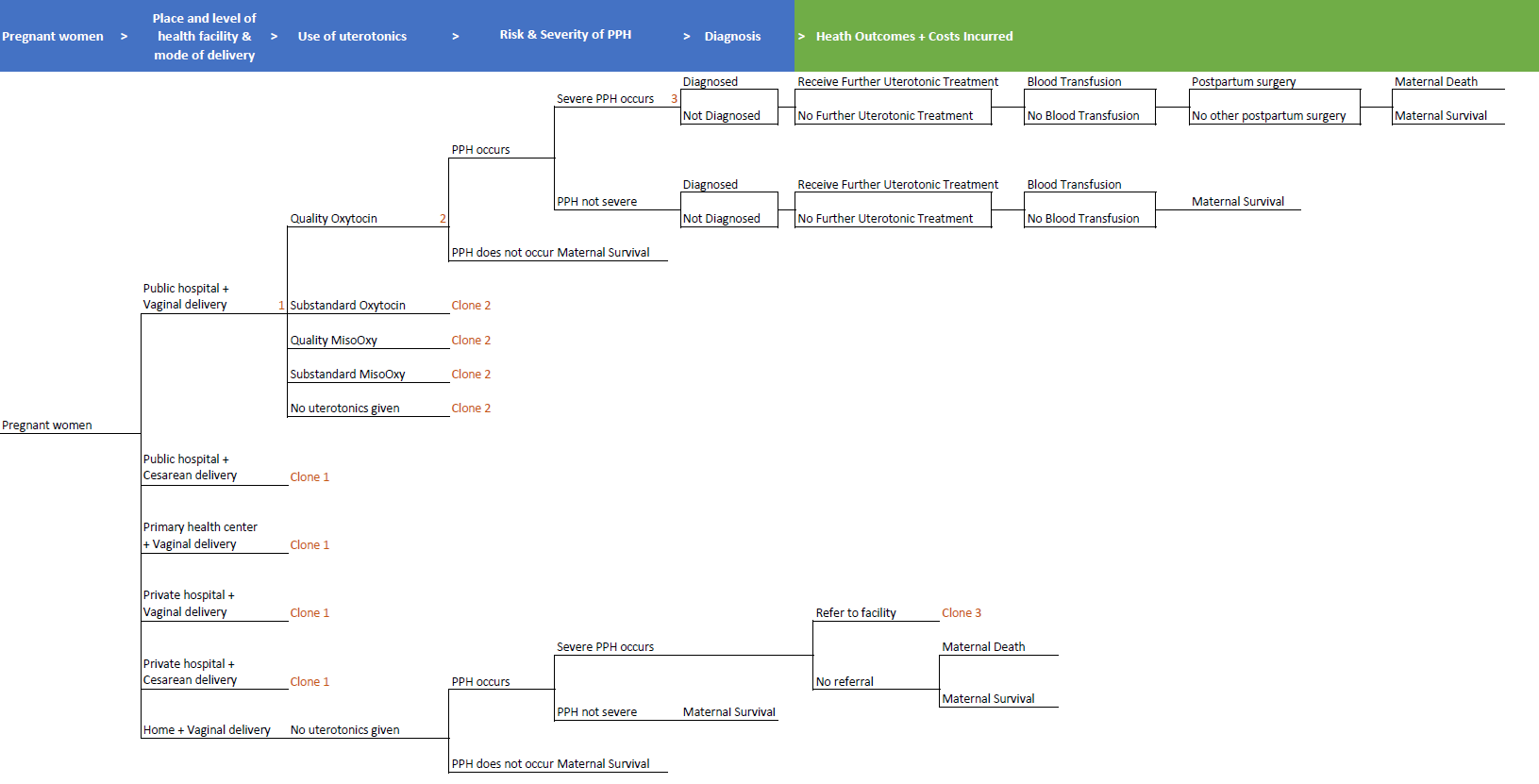


MisoOxy = Misoprostol and Oxytocin; PPH= postpartum hemorrhage.
